# Functional Magnetic Resonance Imaging in Narcolepsy and the Kleine–Levin Syndrome

**DOI:** 10.3389/fneur.2014.00105

**Published:** 2014-06-25

**Authors:** Maria Engström, Tove Hallböök, Attila Szakacs, Thomas Karlsson, Anne-Marie Landtblom

**Affiliations:** ^1^Division of Radiological Sciences, Department of Medical and Health Sciences (IMH), Linköping University, Linköping, Sweden; ^2^Center for Medical Image Science and Visualization (CMIV), Linköping University, Linköping, Sweden; ^3^Department of Pediatrics, Institute of Clinical Sciences, Sahlgrenska Academy, University of Gothenburg, Gothenburg, Sweden; ^4^Department of Pediatrics, Halmstad County Hospital, Halmstad, Sweden; ^5^Division of Disability Research and Linnaeus Centre HEAD, Department of Behavioral Science and Learning, Linköping University, Linköping, Sweden; ^6^Department of Neurology, Department of Clinical and Experimental Medicine (IKE), Linköping University, Linköping, Sweden; ^7^Department of Medical Specialist, Department of Medicine and Health Sciences, Linköping University, Motala, Sweden; ^8^Department of Neuroscience, Uppsala University, Uppsala, Sweden

**Keywords:** functional magnetic resonance imaging, narcolepsy, hypersomnia, Kleine–Levin syndrome, sleep, ascending arousal system, hypothalamus, thalamus

## Abstract

This work aims at reviewing the present state of the art when it comes to understanding the pathophysiology of narcolepsy and the Kleine–Levin syndrome (KLS) from a neuroimaging point of view. This work also aims at discussing future perspectives of functional neuroimaging in these sleep disorders. We focus on functional magnetic resonance imaging (fMRI), which is a technique for *in vivo* measurements of brain activation in neuronal circuitries under healthy and pathological conditions. fMRI has significantly increased the knowledge on the affected neuronal circuitries in narcolepsy and the Kleine–Levin syndrome. It has been shown that narcolepsy is accompanied with disturbances of the emotional and the closely related reward systems. In the Kleine Levin syndrome, fMRI has identified hyperactivation of the thalamus as a potential biomarker that could be used in the diagnostic procedure. The fMRI findings in both narcolepsy and the Kleine–Levin syndrome are in line with previous structural and functional imaging studies. We conclude that fMRI in combination with multi-modal imaging can reveal important details about the pathophysiology in narcolepsy and the Kleine–Levin syndrome. In the future, fMRI possibly gives opportunities for diagnostic support and prediction of treatment response in individual patients.

## 1 Introduction

Narcolepsy and the Kleine–Levin syndrome (KLS), a specific form of periodic idiopathic hypersomnia, are two different sleep disorders characterized by excessive sleepiness. However, the temporal course of the sleepiness and concomitant neurological symptoms are different. Although, significant progress regarding the etiology of KLS and especially narcolepsy has been made recently, the pathophysiological mechanisms underlying the diversity of symptoms in these disorders are not fully defined.

### 1.1 Narcolepsy: Symptoms and etiology

Narcolepsy is characterized by excessive daytime sleepiness, nocturnal dysomnia, and cataplexy (1). The excessive daytime sleepiness is manifested as abruptly impinging sleep attacks at inactivity. Cataplexy is a sudden muscle weakness that often is initiated by positive emotion and laughter. Other typical features are sleep onset rapid eye movement (SOREM), hypnagogic hallucinations, and/or vivid dreams. The etiology of narcolepsy is related to hypocretin production in the hypothalamus (2, 3). Hypocretin neurons stabilize sleep-wake states and loss of these neurons results in an imbalance in the regulation of sleep and wakefulness, which leads to narcolepsy (4). Hypocretins are also suggested to regulate emotional processes, which could explain the phenomenon of emotionally triggered cataplexy in narcolepsy (5).

An up to 25-fold increase in the childhood incidence of narcolepsy has recently been described in relation to the 2009 H1N1 pandemic vaccination campaign in Finland and Sweden (6–9). In China, an increase in narcolepsy was observed after the H1N1 influenza itself, independent of H1N1 vaccination (10). A combination of genetic and environmental factors has been hypothesized to be involved in the pathogenic mechanisms of narcolepsy, where an autoimmune process triggered by seasonal *Streptocoocus*, H1N1 infection, and following AS03-adjuvanted pH1N1 influenza vaccination is leading to hypothalamic destruction with loss of hypocretin neurons (11). Very recently, it was discovered that narcolepsy patients have T cells that are reactive to hypocretin, supporting that narcolepsy is an autoimmune disease (12). The autoimmune hypothesis is also supported by recent findings of reduced levels of beta-amyloid in CSF in narcolepsy (13).

### 1.2 KLS: Symptoms and etiology

KLS is another disorder characterized by excessive sleepiness, but with different characteristics as compared to narcolepsy. KLS is characterized by extremely long sleep periods or prolonged night-time sleep. These periods of excessive sleepiness typically last about one to two weeks and recur several times a year (14, 15). In contrast to narcolepsy, KLS is a relapsing disorder with mean duration of 14 years. In addition to excessive sleepiness, the KLS patients also have cognitive (working memory, language), perceptual (delusions, hallucinations), and behavioral disturbances (binge eating, hypersexuality) during their sleep episodes. Between sleep episodes, the patients have normal sleep and absence of the other accompanying symptoms. However, persistent working memory problems have been reported between sleep episodes (16) as well as after relapse (17).

The etiology of KLS is to a large extent unknown and remains a challenge. Hypothalamic abnormality has been suggested due to the important function of the hypothalamus in sleep regulation. Hypoperfusion or decreased metabolism have been observed in the hypothalamus in a few cases (18–21), however, hormone and hypocretin levels are usually normal. Interperiodic hypoperfusion in cerebral blood flow (CBF) demonstrated by single photon emission tomography (SPECT) is also described in frontal and temporal regions (17, 22). In addition, several functional neuroimaging studies have indicated complex involvement of thalamo-cortical circuits in KLS (See Engström (23) and Arnulf et al. (24) for reviews).

### 1.3 Aim

We aim to show if pathophysiological mechanisms in narcolepsy and KLS could be revealed by *in vivo* measurements and visualization of anatomical biomarkers of brain function. This will be done by reviewing the functional magnetic resonance imaging (fMRI) literature on narcolepsy and KLS. We will also discuss future possibilities to further investigate brain pathology in narcolepsy and KLS using novel fMRI and multi-modal methods.

## 2 Functional Magnetic Resonance Imaging

fMRI records the blood oxygen level dependent (BOLD) signal, which is a response to neural activation in the brain. The BOLD response reflects local changes in deoxygenated hemoglobin (Hb) concentration as deoxygenated Hb has different magnetic properties compared to oxygenated Hb, this leads to a change in the MR signal (25). The biophysical mechanisms behind the BOLD response, however, remains elusive. Several models describing oxygen metabolism and hemodynamics have been proposed (26). Recent research suggests that neurotransmitters directly cause the BOLD response via signaling pathways in glia cells (27).

Traditionally, the BOLD response in fMRI is measured as the neurovascular response to cognitive tasks or sensory stimuli. Thus, fMRI experiments consist of extrinsic stimuli presented to the participant within pre-defined time intervals; so called fMRI paradigms. During image analysis, the correlation between the time course of the paradigm and the time course of the BOLD response is calculated (28). If there is a significant correlation between the paradigm and the BOLD time course in a certain region of the brain, this region is said to be activated by the task. The advantage with task-related fMRI is that detected BOLD responses could directly be related to a certain cognitive task or sensory stimulus. On the other hand, the examination is of limited use when experiments involve sleeping participants.

If active participation is unattainable or unwanted, examination during resting wakefulness or sleep without specific tasks or stimuli is possible. This because brain regions that are involved in cognitive tasks or respond to sensory stimuli display coherent low frequency BOLD fluctuations also without any task or stimuli administered (29). This phenomenon was first discovered in the so-called default mode network (DMN), which is consistently more activated during resting control conditions compared to any fMRI task. Later, it was discovered that DMN also show coherent BOLD signal fluctuations when no tasks or stimuli were present (30–33). It has now been shown that several other networks of the brain, for example the executive and the salience networks, also present coherent fluctuations during resting wakefulness. Furthermore, combining fMRI with EEG, electrooculogram (EOG), and/or video recordings provides excellent opportunities to study event-related brain oscillations (34–36) and functional connectivity during different sleep-stages (37, 38). Thus, both spontaneous and sleep-stage related brain oscillations can be studied using fMRI.

## 3 fMRI in Narcolepsy

fMRI studies in narcolepsy patients with cataplexy demonstrate abnormal processing in the brain’s emotional networks. Two independent studies in 10 and 12 narcolepsy patients, respectively, used a similar paradigm with humorous and not humorous or neutral pictures (39, 40). In both studies, the narcolepsy patients showed increased BOLD responses to humorous stimuli in the amygdala, nucleus accumbens, and the insula compared with control subjects. However, the BOLD responses to humorous stimuli in the hypothalamus were opposite in these two studies: Schwartz and co-workers (40) found increased hypothalamic response in the control group, whereas Reiss and co-workers (39) found increased hypothalamic response in the narcolepsy group. In another study on 14 narcolepsy–cataplexy patients, Ponz et al. (5) observed that narcolepsy patients had abnormal activation in the reward system using the monetary incentive delay task (MID). The MID task evokes BOLD responses to the experience of monetary gains and losses. In this study, the authors observed increased cortical activity in the amygdala and the dorsal striatum for positive gaming outcomes in narcolepsy patients compared to controls. In yet another study, the same authors reported reduced activation in the amygdala in narcolepsy–cataplexy during aversive conditioning (41). In the latter study, the aversive conditioning was induced by a brief painful electrical stimulation.

From these four previous fMRI studies, it can be concluded that narcolepsy with cataplexy is associated with abnormal BOLD responses in the emotional and the closely related reward systems. It appears that positive stimulation is associated with increased BOLD responses in the amygdala, whereas negative stimulation is associated with decreased responses. However, the BOLD response to humorous stimuli in the hypothalamus remains elusive since two studies found contradictory results. Nevertheless, these fMRI findings are in line with structural neuroimaging, which have shown narcolepsy-related aberrations in brain structures, such as the amygdala, nucleus accumbens, midbrain, thalamus, hippocampus, and fronto-temporal cortical areas (42–45).

Only few fMRI studies have investigated the relation between brain activation and pharmacological therapy in narcolepsy (46–48). Because of the low number of subjects and the outdated imaging techniques in the older studies, these results remain inconclusive.

## 4 fMRI in KLS

During periods of hypersomnia, KLS patients are frequently affected by cognitive disturbances, of which working memory problems are common (14, 15). Our group has previously reported that working memory dysfunction may persist between the periods of hypersomnia (17). The finding of working memory dysfunction in KLS was further investigated by fMRI aiming to describe the neural networks involved (16, 49, 50). During fMRI, KLS patients and healthy controls were administered the Daneman and Carpenter reading span task, which is a complex working memory task (51). The task was designed with four difficulty levels and data was analyzed for parametric responses to increased cognitive demand.

**Figure 1:**
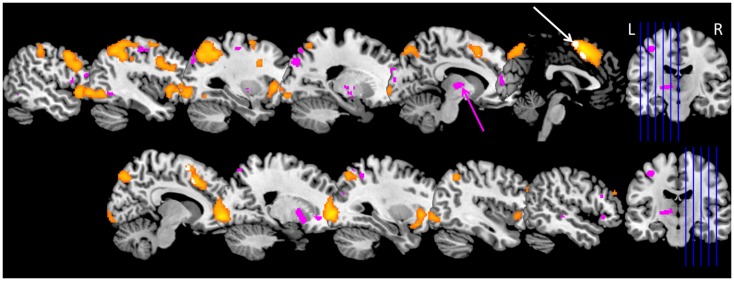
**Brain activation in Kleine–Levin syndrome (KLS) during working memory performance**. The orange areas show typical working memory activation in the executive network in healthy individuals. Pink areas show regions with hyperactivation in KLS patients. The main finding of thalamic hyperactivation is marked with a pink arrow. The white arrow marks areas within the orange activation cluster in the anterior cingulate cortex and adjacent medial prefrontal cortex where KLS patients had less activation compared to the healthy control group. Data include 18 KLS patients and 18 matched controls (23).

During the complex working memory task, KLS patients show hyperactivation in the left thalamus as compared to healthy controls. They also show less activation in the anterior cingulate cortex and adjacent medial prefrontal cortex (16). These results could be reproduced in a larger patient group and also in patients who returned for a second examination (23, 49, 50). An overview of the results for the extended study, including 18 KLS patients and 18 controls, is shown in Figure 1. Furthermore, KLS patients had lower working memory capacity; both in the task administered during fMRI and in the standard version of the listening span task. These findings demonstrate that KLS patients have deteriorated working memory function that affects thalamo-cortical networks also between periods of hypersomnia. A subsequent study aimed to describe working memory function in KLS on a network level by investigating the functional connectivity (49). KLS patients seemed to have non-optimal recruitment of neural resources in the executive network during task performance, and they also had increased functional connectivity between the left thalamus and the executive and salience networks, respectively.

The consistent findings of hyperactivation of the thalamus during fMRI indicate a possible thalamic pathology in KLS. We therefore measured the concentration of pathology biomarkers using magnetic resonance spectroscopy (MRS) (50). However, MRS data showed no difference between KLS patients and controls regarding any of the investigated biomarkers. On the other hand, there was a significant negative correlation between the level of brain activation during working memory performance and the concentration of *N*-acetylaspartate (NAA) in the left thalamus. This finding indicates a possible neurodegenerative process in KLS patients with high thalamic activation.

The most consistent fMRI finding in KLS is abnormal thalamic function (52). Thalamic abnormality is also reported from SPECT studies investigating cerebral perfusion in KLS. Huang et al. reported hypoperfusion of the left thalamus in 18 out of 30 KLS patients and of the right thalamus in three patients (53). In contrast to fMRI, which was performed in the asymptomatic state between sleep episodes, hypoperfusion of the thalamus was reported in KLS patients during sleep episodes (19, 54, 55). Thus, imaging thalamic function in individual patients could provide biomarkers of KLS pathology. In a recent study, using binary logistic regression, we found that fMRI of the thalamus and assessment of working memory performance predicted KLS diagnosis with 80% accuracy when comparing with healthy subjects (23).

## 5 Future Perspectives: Imaging Biomarkers of Narcolepsy and KLS

### 5.1 Anatomical biomarkers of brain function

**Figure 2:**
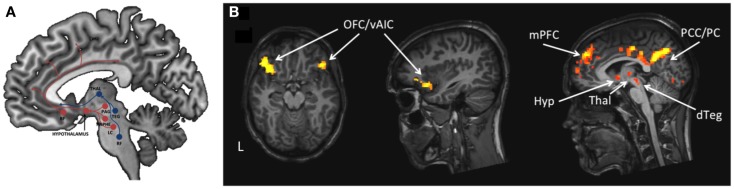
**The ascending arousal system involved in regulation of sleep and wakefulness**. **(A)** The figure is a schematic description of the ventral (red) and dorsal (blue) pathways of the ascending arousal system. BF = basal forebrain, Thal = thalamus, PAG = periaqueductal gray, Raphe = Raphe nuclei, LC = locus coeruleus, TEG = tegmentum, RF = reticular formation. **(B)**. The figure shows brain activation in one male narcolepsy patient during emotional flanker task (57) in emotional and sleep regulating networks. OFC = orbitofrontal cortex, vAIC = ventral anterior insular cortex, mPFC = medial prefrontal cortex, PCC = posterior cingulate cortex, PC = precuneus, Hyp = hypothalamus, Thal = thalamus, dTEG = dorsal tegmentum.

By fMRI it is possible to identify the anatomical location of neural entities that are involved in different aspects of brain function related to narcolepsy and KLS and their concomitant symptoms, as shown in the literature review. Here, in Figure 1, we visualize thalamic hyperactivity in KLS during working memory performance (16, 23, 49). We also show preliminary results of brain activation in emotional (56) and sleep regulating networks (4) in a patient with narcolepsy (Figure 2); results that are in agreement with previous literature (5, 39–41). Thus, the dorsal branch of the ascending arousal system including the thalamus is presumably affected in KLS and the ventral branch including the hypothalamus in narcolepsy (Figure 2). Based on findings in the literature, we argue that fMRI has the potential to image anatomical biomarkers of brain function in narcolepsy and KLS, and possibly also in other disorders of pathological sleep. In KLS, it has been shown that fMRI measures of thalamic activation combined with working memory assessment could predict diagnosis with high accuracy when comparing with healthy individuals (23). Thus, fMRI in KLS could possibly be used as diagnostic aid. This finding must, however, be validated by comparing fMRI in KLS and other differential diagnosis. In narcolepsy, fMRI has been used to investigate the relation between brain activation and pharmacological therapy (46–48). Although results were promising, they remain hypothetical until validated in larger patient groups. Improved methods for data acquisition and analysis are necessary to fully utilize the potential of fMRI for diagnosis and prediction of treatment response in individual patients.

### 5.2 Dynamic biomarkers of brain function

Dynamic biomarkers describe the temporal courses of brain function in regions of interest or interactions within brain networks, so called functional connectivity. Powerful methods for brain connectivity assessment, such as dynamic causal modeling (DCM) (58), reveal mechanisms of actions in brain networks. By DCM, neural interactions between the thalamus and the prefrontal cortex, and their modulation by working memory performance, can be studied in KLS. In narcolepsy, DCM can be used to study neural interactions between the hypothalamus and the amygdala and their modulation by emotional processing. In previous studies, it has been shown that DCM is powerful when investigating pharmacological treatment responses in the brain (59, 60). A completely new approach to study brain dynamics is mechanistic modeling of the event-related BOLD response using ordinary differential equations (61). By this method it is possible to study the underlying neurovascular dynamics of the BOLD response, e.g., neurotransmitter release and the effect of vasoactive agents (27).

### 5.3 Multi-modal imaging

To further advance the understanding of brain function in narcolepsy and KLS, fMRI could preferentially be combined with other imaging methods, so called multi-modal imaging. Positron emission tomography (PET) has provided valuable information about pathology in narcolepsy and KLS (62–65). The multitude of imaging protocols for structural and functional imaging makes MRI a multi-modal method itself. In MRI, quantitative relaxometry (66) or diffusion tensor imaging (DTI) can give information about pathological changes in brain microstructure. Thus, these methods can be used to investigate possible microstructure changes in the thalamus in KLS or pathological white matter integrity in the emotional system in narcolepsy. By magnetic resonance spectroscopy (MRS), the concentration of different metabolites in the brain has been measured in narcolepsy (67, 68). Novel images sequences allow measurements of the absolute concentrations of the neurotransmitters GABA and glutamate (69). Information about neurotransmitter levels could be used as input in the mechanistic models discussed above (61), and brain GABA concentration could be related to CSF orexin levels to further the understanding of pathophysiology in narcolepsy.

Previous fMRI studies on narcolepsy and KLS were performed during wakefulness. No fMRI studies have so far looked at sleep and sleep dysfunction in these sleep disorders. By simultaneous acquisition of fMRI and EEG data, it is possible to explore the dynamics of brain function during different sleep-stages. Previous fMRI–EEG studies in healthy individuals show specific brain activation patterns related to characteristic sleep-stages, such as slow wave (SW) and rapid eye movement (REM) sleep, as well as brain activity related to sleep spindles and K-complexes (34, 36–38). Thus, combined fMRI–EEG would certainly add important information about the pathophysiology in narcolepsy and KLS by investigating the typical SOREM sleep in narcolepsy or the hypersomnic state in KLS in comparison to normal sleep.

### 5.4 Ultra-high resolution imaging of brain function

While fMRI has revolutionized the understanding of the human brain during the last 10 years, its spatial resolution, which today is in the millimeter range, is far from what is possible, and its temporal resolution is much slower than the speed of neural processes. Ultra-high field (UHF) MRI with magnetic field strengths above 7T would improve the sensitivity of fMRI dramatically. However, when UHF-fMRI was introduced, there was a concern about increased risk of susceptibility artifacts in regions adjacent to nasal and oral cavities. Recent studies have now demonstrated improved detection of brain activation in ventral brain regions, such as the amygdala, and also in subcortical structures (70, 71). Since the amygdala is a key region of interest in narcolepsy and the ascending arousal system has important subcortical hubs (Figure 2), this research indicates that it is possible to obtain more detailed information about the brain function in areas related to sleep pathology using UHF-fMRI.

## 6 Conclusion

By fMRI, it is possible to identify anatomical biomarkers of brain function related to narcolepsy and KLS and their concomitant symptoms. However, the clinical use of fMRI must be validated in future studies. Novel fMRI and multi-modal methods that investigate the dynamics in brain networks involved in sleep regulation should be explored for their potential to investigate pathological sleep. Such technologies are necessary to fully utilize the potential of fMRI for diagnosis and prediction of treatment response in individual patients.

## Author Contributions

All co-authors contributed to the conception and the final revision of the work. Maria Engström made substantial contributions to the design of the work as well as interpretation of the data. Maria Engström drafted and finalized the work, and provided all figures. All co-authors made final approval of the version to be published.

## Conflict of Interest Statement

The authors declare that the research was conducted in the absence of any commercial or financial relationships that could be construed as a potential conflict of interest.
